# Lethality of PAK3 and SGK2 shRNAs to Human Papillomavirus Positive Cervical Cancer Cells Is Independent of PAK3 and SGK2 Knockdown

**DOI:** 10.1371/journal.pone.0117357

**Published:** 2015-01-23

**Authors:** Nannan Zhou, Bo Ding, Michele Agler, Mark Cockett, Fiona McPhee

**Affiliations:** 1 Department of Virology, R&D of Bristol-Myers Squibb Company, Wallingford, Connecticut, United States of America; 2 Department of Leads Discovery, R&D of Bristol-Myers Squibb Company, Wallingford, Connecticut, United States of America; Georgetown University, UNITED STATES

## Abstract

The p21-activated kinase 3 (PAK3) and the serum and glucocorticoid-induced kinase 2 (SGK2) have been previously proposed as essential kinases for human papillomavirus positive (HPV+) cervical cancer cell survival. This was established using a shRNA knockdown approach. To validate PAK3 and SGK2 as potential targets for HPV+ cervical cancer therapy, the relationship between shRNA-induced phenotypes in HPV+ cervical cancer cells and PAK3 or SGK2 knockdown was carefully examined. We observed that the phenotypes of HPV+ cervical cancer cells induced by various PAK3 and SGK2 shRNAs could not be rescued by complement expression of respective cDNA constructs. A knockdown-deficient PAK3 shRNA with a single mismatch was sufficient to inhibit HeLa cell growth to a similar extent as wild-type PAK3 shRNA. The HPV+ cervical cancer cells were also susceptible to several non-human target shRNAs. The discrepancy between PAK3 and SGK2 shRNA-induced apoptosis and gene expression knockdown, as well as cell death stimulation, suggested that these shRNAs killed HeLa cells through different pathways that may not be target-specific. These data demonstrated that HPV+ cervical cancer cell death was not associated with RNAi-induced PAK3 and SGK2 knockdown but likely through off-target effects.

## Introduction

Human papillomaviruses (HPVs) are small DNA tumor viruses that infect cutaneous or mucosal epithelial cells [1]. To date, 170 HPV types have been fully characterized, and approximately 40 types infect the genital tract [2]. The genital HPV types are sexually transmitted and can be further divided into low-risk and high-risk groups according to the propensity of their induced lesions to progress to malignancy. Persistent high-risk human papillomavirus (HPV) infection is the major cause of cervical cancer. Once integrated into the host genome, high-risk HPV types exert their oncogenic effects primarily through the continuous expression of the oncoproteins E6 and E7 [3]. Many activities have been described for both of these oncoproteins, among which the following are best characterized and critical for transformation: E6 binds to E6-associated protein (E6-AP) resulting in the ubiquitination and degradation of tumor suppressor protein p53; E7 binds to pocket protein family members, in particular, the retinoblastoma protein (Rb) causing inactivation and degradation of Rb [4]. Interactions between high-risk HPV oncoproteins and endogenous cellular proteins have been shown to trigger cell cycle deregulation and apoptosis, and a subsequent increase in the replication of transformed cells, progressing to cancer [5].

RNA interference (RNAi) has become a widely used tool for functional genomic studies in vertebrates and invertebrates [6]. RNAi works by silencing a gene through homologous short interfering double-strand RNAs (siRNAs), which trigger the destruction of corresponding messenger RNA (mRNA) by the RNA-induced silencing complex (RISC) [7]. The ease, speed, and cost-effectiveness have made it the method of choice for loss-of-gene function studies. Recently, high-throughput RNAi screens were used to explore the differences in kinase requirements for proliferation and survival among various cancer cells [8–10]. A common set of kinases were observed as being required for proliferation/survival of three cervical carcinoma cell lines (CaSki, HeLa and SiHa) but dispensable for primary human foreskin keratinocytes (HFKs). It was proposed that the p21-activated kinase 3 (PAK3) and the serum and glucocorticoid-induced kinase 2 (SGK2) were essential for HPV positive (HPV+) cervical cancer cell survival. The lethality caused by SGK2 or PAK3 depletion in HPV E6 expressing cells was a consequence of p53 inactivation [10].

The PAK proteins are serine/threonine kinases and divided into two groups. Group I PAKs includes PAK1 through 3; these kinases bind to and are catalytically activated by Rac and cdc42 GTPases [11, 12]. PAK3 is abundantly expressed in the central nervous system (CNS), and is specifically implicated in neuronal plasticity and spinogenesis [13]. PAK3 also regulates cell cycle progression, neuronal migration and apoptosis [13–16]. Loss of function of PAK3 is responsible for X-linked non-syndromic mental retardation [17, 18]. The SGK family of kinases includes SGK1 through 3; SGK2 is the most poorly studied member of this family. Unlike SGK1, SGK2 mRNA is not induced by stimulation with serum or glucocorticoid, and is only present at significant levels in liver, kidney and pancreas and at lower levels in the brain [19]. However, similar to SGK1 and 3, SGK2 also activates certain potassium and sodium channels, suggesting an involvement in the regulation of processes such as cell survival, neuronal excitability, and renal sodium excretion [20, 21].

Specific annihilation of cervical cancer cells would be of significant interest to the anti-cancer research community. To confirm that blocking the function of SGK2 or PAK3 by a p53-dependent pathway is associated with HPV+ cell depletion, appropriate controls are essential when using an RNAi approach. Target specificity has been a source of concern since the first application of RNAi to functional genomics. Non-specific effects have been reported that, in addition to the targeted genes, led to changes in the expression of other genes at both the mRNA and protein levels [22–24]. In addition, induction of genes involved in the interferon response machinery has been observed [25–28], further challenging the reliability of RNAi in loss-of-function studies. In this study, we demonstrate that the phenotypes of HPV+ cervical cancer cells induced by PAK3 or SGK2 shRNAs could not be rescued by complement expression of respective cDNA constructs. The HPV+ cervical cancer cells are also susceptible to several non-human target shRNAs. These data show that the loss of viable HPV+ cervical cancer cells does not correlate with RNAi-induced PAK3 and SGK2 knockdown, as previously reported [10].

## Materials and Methods

### Cells

HeLa (ATCC CCL-2) and CaSki (ATCC CRL-1550) cells were grown in Dulbecco′s Modified Eagle Medium (DMEM) supplemented with 1% Penicillin-streptomycin and 10% fetus bovine serum.

### Plasmids

Glycerol stocks of bacteria or plasmids harboring PAK3, SGK2 and control (Luciferase, EGFP, Turbo-GFP and Non-target) Mission shRNA lentivirus vectors (Table 1) were purchased from Sigma (St. Louis, MO). The PAK3and SGK2shRNA expression cassettes in a lentivirus backbone were characterized and validated previously by the RNAi Consortium (TRC, Broad institute, Cambridge, MA). Plasmids were purified using the Endo-free Maxi plasmid kit (Qiagene, Valencia, CA) according to the manufacturer’s protocol. The doxycycline (dox)-inducible pLKO-Tet-On plasmid was a gift from Dr. Susan Wee [29]. ShRNA sequences from the Mission shRNA lentivirus vectors (Table 1) were inserted between AgeI/EcoRI sites of the pLKO-Tet-On using the standard cloning technology.

**Table 1:** Study shRNA lentivirus vectors.

**Target gene**	**shRNA**	**The RNAi Consortium Number**	**Abbreviation**	**Targeting region**
PAK3	PAK3	TRCN0000003242	3242	Coding sequence
		TRCN0000003244	3244	Coding sequence
		TRCN0000003245	3245	Coding sequence
		TRCN0000195142	5142	Coding sequence
		TRCN0000199628	9628	Coding sequence
SGK2	SGK2	TRCN0000002110	2110	Coding sequence
		TRCN0000002111	2111	Coding sequence
		TRCN0000002112	2112	Coding sequence
		TRCN0000002113	2113	3′UTR
Luciferase	Luc	NA^a^	Luc	Coding sequence
EGFP	EGFP	NA	EGFP	Coding sequence
Turbo-GFP	T-GFP	NA	T-GFP	Coding sequence
Non-Target	NT	NA	NT	Coding sequence

^a^ NA—not available

To construct a knockdown-deficient control PAK3 shRNA, a range of shRNA 3245 mutants with different mismatched nucleotides to the target mRNA were designed and cloned to pLKO-Tet-On vector. After quantifying the decrease of PAK3 mRNA copy number by these mismatching shRNAs, one knockdown-deficient PAK3 shRNA (shRNA 3245 m15) with a single mismatch at position 15 (CCAAACTTCCAACAgAACA) was identified.

Ultimate ORF human clones for PAK3 (NCBI Reference Sequence: NM_002578.3), and SGK2 variants 1 and 2 (NM_170693.1 and NM_016276.3) were purchased from Invitrogen (Carlsbad, CA). ORF sequences of PAK3 and SGK2 were introduced into the expression vector pcDNA3.2-Dest using the Gateway technology (Invitrogen). For rescue experiments, PAK3 and SGK2 mutant-expressing plasmids were constructed harboring 4 to 5 silent mutations in the shRNA complementary sequences. Mutations were introduced into pcDNA-PAK3 using the QuikChange site-directed mutagenesis kit (Agilent Technologies, Santa Clara, CA) and the following primer pairs: pPAK-3244mut 5’-GATAGCTACTTGGTGGGTGACGAGTTGTGGGTAGTCATGGA ATACTT-3’ and 5’-AAGTATTCCATGACTACCCACAACTCGTCACCCACCAAGT AGCTATC-3; pPAK3–3245mut 5’-GGCACGATTACTCCA AACCAGCAATATAACA AAATTGGAACAG-3’ and 5’-CTGTTCCAATTTTGTTAT ATTGCTGGTTTGGAGT AATCGTGCC-3; pPAK3–5142mut 5’-GTTGATATCTGGTCTCTTGGCATCATGGCC ATCGAAATGGTGGAAGGTGAACC-3’ and 5’-GGTTCACCTTCCACCATTTCGAT GGCCATGATGCCAAGAGACCAGATATCAAC-3’. Mutations were introduced into pcDNA-SGK2 using the following primer pairs: pSGK2–2111mut 5’-GTAGAGCCTGA AGACACCACCAGCACCTTCTGTGGTACCCCTGA GT-3’ and 5’-ACTCAGGGGTA CCACAGAAGGTGCTGGTGGTGTCTTCAGGCTCT AC-3’; pSGK2–2112mut 5’-CA TTGGCTACCTGCACT CCTTGAATATTATTTACAG GGATCTGAAACC-3’ and 5’-GGTTTCAGATCCCTGTAAATAATGTTAAGTGAGTGCAGGTAGCCAATGGCG-3’. These plasmids were confirmed as being refractory to PAK3 or SGK2 shRNA knockdown using qPCR for mRNA quantification and Western immunoblot for protein quantification.

### Lentivirus production

Recombinant lentiviruses were produced by cotransfecting Lenti-X 293T cells (Clontech, Mountain View, CA) with the shRNA lentivirus vector, packaging plasmid and the VSV-G expressing plasmid. Briefly, 3 × 10^6^ Lenti-X cells were seeded in a 10 cm dish prior to transfection. For each 10 cm dish, 3 μg shRNA-lentivirus plasmid, 2.7 μg pCMVΔ8.9 (packing plasmid), and 0.3 μg pVSV-G were mixed with TransIT-293 reagent (Mirus Bio, Madison, WI) in Opti-MEM and incubated at room temperature for 20 minutes. Transfection mixture was added to the cells and incubated overnight. The medium was aspirated and fresh medium added to support virus growth. The culture medium containing infectious lentivirus was collected 48 h after transfection, centrifuged to remove cell debris, and filtered through a 0.45 μm filtration unit. Virus was titered with a p24 assay kit (Perkin Elmer, Waltham, MA) and puromycin selection method recommended by Sigma (www.sigmaaldrich.com). Viral titers of ~ 3 × 10^7^ CFU/ mL were obtained using this method; virus yield was similar, irrespective of the input shRNA expression vectors.

### Cell viability/proliferation assay

Cells were seeded in 96-well plates at 2000 cells/well in 100 μL of medium prior to virus infection. The infectious shRNA lentivirus was diluted in medium containing 10 μg/mL polybrene. Medium was removed from the cells, and serial dilutions of 100 μL of lentivirus were added to each well. After 24 hours, virus was removed and replaced with 100 μL of fresh medium. For experiments using the inducible shRNAs, fresh medium containing 20 ng/mL doxycycline was added. Cell viability was measured 5 days after infection using the CellTiter-Blue reagent (Promega, Madison, WI). The CellTiter-Blue reagent was diluted at a ratio of 1:4 in medium, and 20 μL of the diluted reagent was added to each well and incubated at 37°C for 2 hours before recording fluorescence (531_Ex_/595_Em_) on an Envision 2104 multilabel reader (Perkin Elmer, Waltham, MA). Live cells were counted using a Guava ViaCount assay as described in the manufacturer’s protocol (EMD Millipore, Billerica, MA).

### Cell apoptosis assay

Seeding and infection of cells were performed as described for the cell proliferation assay except that 80 μL of medium/well was added after the virus was discarded. An apoptosis assay was performed 72 hours after infection using a caspase 3/7 glo assay kit (Promega, Madison, WI). Briefly, the substrate of caspase 3/7 was diluted in lysis buffer and 20 μL of the diluted substrate was added to each well. The plate was incubated at room temperature for 1 hour and the luminescence signal read on an Envision multi-label reader.

### Cell autophagy assay

HeLa cells were infected with serially diluted lentiviral SGK2 shRNA as well as with a control non-target (NT) shRNA (Table 1). Virus was removed after 24 hours of infection and cells were cultured for an additional 48 hours. Rapamycin (1 μM, EMD Chemicals, Gibbstown, NJ) was included as a control on each plate and added 24 hours before the plates were processed for imaging. Cell plates were fixed by the addition of 20 μl of fixation buffer [(4% v/v formaldehyde in Dulbecco′s Phosphate-Buffered Saline (DPBS)] directly to the assay medium and incubated for 15 minutes at room temperature followed by 2 washes with DPBS. The cells were immunostained for induction of autophagy by permeabilization for 10 minutes with 0.1% Triton X-100 in a 1% bovine serum albumin (BSA)/DPBS buffer. A blocking buffer of 5% fetal bovine serum (FBS) in DPBS was used during antibody incubations which consisted of a LC3B primary antibody (Cell Signaling Technologies, Danvers, MA) followed with an Alexa 488-conjugated secondary antibody (Invitrogen, Carlsbad, CA). The nucleus was stained with Hoescht dye (Invitrogen, Carlsbad, CA) and actin was stained with Alexa 647-phalloidin (Invitrogen, Carlsbad, CA) to identify the cell.

Images were acquired on the confocal Opera High Content Imager (Perkin Elmer, Watham, MA). A 20x water immersion objective (20x water_Lucplfln NA = 0.7), was used with a 488 diode laser (excitation filter: λ = 520 nm, 75 nm bandpass), a non-confocal UV light source (excitation filter λ = 450 nm, 50 nm bandpass) and a 640 diode laser (excitation filter: λ = 690, 50 nm bandpass). For each experimental condition, 12 fields/well (~1200 cells) were acquired and analyzed using a modified Acapella (Perkin Elmer) algorithm measuring the intensity of LC3B staining in the cytoplasm area normalized on a per cell basis.

### Phenotype rescue

Cells were seeded at 2 × 10^5^ cells/well in 6-well plates the day before transfection. These cells were transfected with serially diluted PAK3 or SGK2 mutant or wild type-expressing plasmids using Fugene 6 (Roche, Indianapolis, IN) as a transfection reagent. After 6 hours, the transfection medium was removed. Cells were infected with shRNA-expressing lentivirus (1 mL) that had been diluted 1:10 or 1:15 in medium containing 10 μg/mL polybrene, and then incubated at 37°C overnight. Cells were trypsinized and counted, and seeded at 2000 cells/well in 96-well plates. For assays monitoring apoptosis and cell viability, cells were harvested at 72 hours and 5 days after infection, respectively, using the aforementioned methods.

### Quantification of PAK3 and SGK2 mRNAs

Cells were seeded at 2 × 10^5^ cells/well in 6-well plates the day before infection with shRNA-expressing lentivirus. The infection or transfection/infection was performed as previously described. Total RNA was extracted from cells 72 hours after infection using RNeasy Plus Mini Kit plus DNase I digestion (Qiagen, Valencia, CA), according to the manufacturer’s protocol. Extracted total RNA (2–5 μg) was reverse-transcribed in a total volume of 20 μL using SuperScript III First-Strand Synthesis System (Invitrogen, Carlsbad, CA). Resultant cDNA was stored at -20°C until use. PAK3 (Applied Biosystems Taqman Gene Expression Assay ID: Hs00176828_m1) and SGK2 (ID: Hs00367636_m1) assays were performed for quantifying mRNA using a 9700HT Real-time PCR system (Applied Biosystems, Carlsbad, CA). GAPDH mRNA was also measured in the same well to standardize sample loading.

### Quantification of PAK3 and SGK2 proteins

Protein levels were determined from the same transfection and infection experiments used to quantify mRNA knockdown. Cells were harvested 72 hours after infection, washed once with phosphate-buffered saline (PBS), and lysed in lysis buffer (25 mM HEPES, 150 mM NaCl, 1% Igegel CA-630, 0.25% Sodium deoxycholate, 10% Glycerol, 25 mM NaF, 10 mM MgCl_2_, 1 mM EDTA, 1 mM Sodium Vanadate, 1 tablet/50 mL protease inhibitor) on ice for 30 minutes. Lysates were clarified by centrifugation (15,000 rpm) at 4°C for 10 minutes. Total protein, 80 μg, was mixed with a loading buffer and boiled at 100°C for 10 minutes. The samples were subjected to a 4–15% sodium dodecyl sulfate–polyacrylamide gel electrophoresis (SDS–PAGE) and subsequently electrophoretically transferred onto PVDF membranes. After non-specific binding sites were blocked with 5% nonfat milk, the membrane was incubated with primary antibodies (1:1000 dilution; anti-human PAK3 monoclonal antibody N-19 and anti-human SGK2 monoclonal antibody 3Q-2, Santa Cruz Biotechnology, Santa Cruz, CA) at 4°C overnight. The membrane was washed three times and incubated with the respective secondary antibody for 1 hour at room temperature. The immunoreactive bands were visualized with the enhanced chemiluminescence (ECL) reagent according to the manufacturer’s instructions. Protein loading was standardized by including GAPDH protein as a control.

## Results

### RNAi reduces PAK3 and SGK2 expression

**Figure 1:**
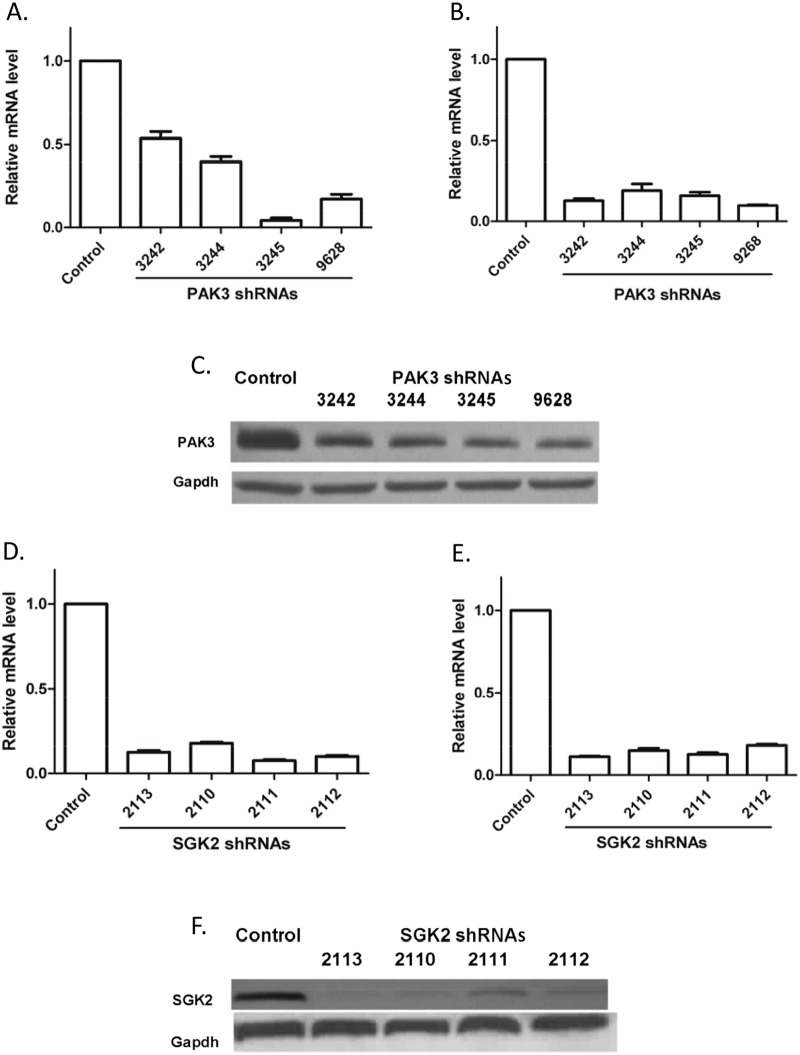
Knockdown of PAK3 and SGK2 by shRNAs. mRNA levels were determined by quantitative reverse transcription PCR analysis at 72 hours after infection with the respective shRNA vectors; control denotes infection with a vector encoding non-target shRNA. Each bar on the bar graph represents the average ± standard deviation of 4 replicates from one of three independent experiments with similar results. mRNA levels were normalized with GAPDH expression; PAK3 and SGK2 protein expression were determined 72 hours after infection using a Western immunoblot. GAPDH protein acted as a protein loading control for each sample. (A) PAK3 shRNA decreased PAK3 mRNA levels in HeLa cells; (B) PAK3 shRNAs reduced PAK3 mRNA expression in DMS-79 cells; (C) PAK3 shRNAs reduced PAK3 protein expression in DMS-79 cells. DMS-79 cell lysates were analyzed with PAK3 monoclonal antibody N-19; (D) SGK2 shRNAs reduced SGK2 mRNA levels in HeLa cells; (E) SGK2 shRNAs reduced SGK2 mRNA levels in GTL16 cells; (F) SGK2 shRNAs reduced SGK2 protein levels in GTL16 cells. GTL16 cell lysates were analyzed with SGK2 monoclonal antibody 3Q-2.

To validate the effect of PAK3 shRNA on PAK3 expression, four independent PAK3 shRNAs were used for targeting different regions of PAK3 mRNA. HeLa cells were infected with lentiviruses harboring the respective PAK3 shRNAs. As a specific control, lentivirus harboring a NT shRNA was employed. The efficiency of lentivirus infection was optimized using a Turbo-GFP expression lentivirus vector; an infection rate of >90% was achieved. A significant knockdown of PAK3 mRNA was observed in HeLa cells after infection with each of the four tested shRNAs (Fig. 1A). Similar results were also observed when using inducible lentivirus expressing PAK3 shRNAs in CaSki cells (data not shown). Since PAK3 protein is expressed at very low levels in HeLa and CaSki cells, and migrates with PAK2, which can also be detected by the N-19 PAK3 antibody (see Materials and Methods), PAK3 protein knockdown was examined in a PAK3 high expression small cell lung carcinoma cell, DMS-79. All of the tested PAK3 shRNAs significantly decreased both PAK3 mRNA and protein levels in DMS-79 cells (Fig. 1B and C).

Similar experiments were performed to validate SGK2 shRNAs. SGK2 mRNA expression in HeLa cells was dramatically reduced by the four SGK2 shRNAs (Fig. 1D) as was SGK2 mRNA expression in CaSki cells by inducible SGK2 shRNAs (data not shown). As with PAK3, SGK2 protein expression is too low to be detected in HeLa and CaSki cells. The knockdown of SGK2 protein was therefore evaluated in GTL16, a SGK2 high expression gastric carcinoma cell line. SGK2 shRNAs significantly reduced SGK2 mRNA as well as protein expression in GTL16 cells (Fig. 1E and 1F). Thus, the knockdown of PAK3 and SGK2 by their corresponding shRNAs was verified.

### Phenotypes induced by PAK3 and SGK2 lentiviral shRNAs

**Figure 2:**
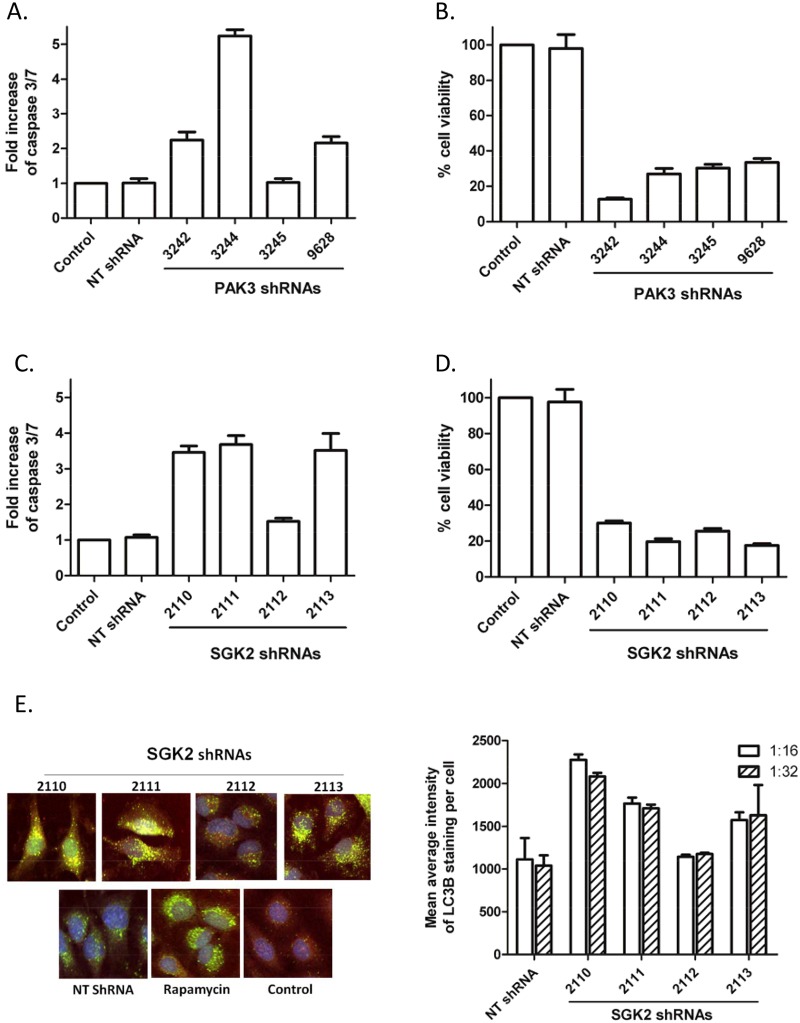
Loss of cell viability induced by PAK3 or SGK2 shRNAs. (A) HeLa cells were infected with PAK3 shRNA at a 1:10 dilution. Cell apoptosis was determined using a caspase 3/7 glo luciferase assay 72 hours after infection. The bar graph presents fold changes of caspase 3/7 activity (luminescence) induced by various shRNAs compared with a no-shRNA control. Data represent the average ± standard deviation of 4 replicates from one of two separate experiments with similar results; (B) Inhibition of proliferation/viability of HeLa cells by PAK3 shRNAs was assessed using a CellTiter blue assay 5 days after infection. The bar graph presents percent viability (fluorescence) of lentiviral shRNA-infected cells compared with a control lentivirus without shRNA expression. Data represent the mean ± standard deviation of three independent experiments; (C) SGK2 lentiviral shRNAs induced HeLa cell apoptosis. Data represent 4 replicates from one of two separate experiments with similar results; (D) SGK2 shRNAs inhibited proliferation/viability of HeLa cells. Data represent the average ± standard deviation of two independent experiments; (E) SGK2 lentiviral shRNAs induced autophagy of HeLa cells. Cells were infected with SGK2 lentiviral shRNAs at 1:16 and 1:32 dilution, respectively. 1 μM Rapamycin was included as an autophagy control on each plate. Cell plates were fixed 72 hour after infection and immunostained for induction of autophagy with a LC3B primary antibody, followed with an Alexa 488-conjugated secondary antibody. Images were captured using the confocal Opera High Content Imager. Images of SGK2 lentiviral shRNA-infected HeLa cells (~1200 cells/well) were captured and cell numbers counted. The average intensity of LC3B staining per cell was measured and calculated using a modified Capella (Perkin Elmer) algorithm. The bar graph presents the average ± standard deviation of LC3B staining intensity derived from two wells.

HeLa cells were infected with PAK3 and SGK2 shRNA-expressing lentiviruses to determine the shRNA-induced phenotypes. CaSki cells were infected with inducible PAK3 and SGK2 shRNA expressing lentiviruses. The NT shRNA and a lentivirus vector containing no shRNA expression cassette were used as controls. For the phenotype study, lentivirus infection with and without centrifugation were compared. Although better infection efficiency can be achieved by the spin-inoculation with the same amount of virus, the toxicity associated with NT shRNA and even the no shRNA virus control increased proportionally (data not shown). Therefore, cells were infected without centrifugation. Cells were split in different plates 24 hours after infection for caspase 3/7 and cell proliferation/viability assays. A time course of caspase 3/7 expression induced by the various PAK3 shRNAs indicated peak expression 72 hours after infection (Fig. 2A; time course data not shown). Both PAK3 and SGK2 shRNAs reduced HeLa and CaSki cell viability (Fig. 2B and 2D, data not shown for CaSki cells) as previously reported [10]. The four PAK3 shRNAs varied in their capacity to induce HeLa cell apoptosis (Fig. 2A). The shRNA, 3244, was the most potent at triggering apoptosis, whereas shRNA 3245 displayed marginal induction of apoptosis, despite its ability to strongly suppress PAK3 mRNA in HeLa cells (Fig. 1A). In addition, shRNA 3245 inhibited cell proliferation/viability similarly to the strongest apoptosis inducer, shRNA 3244 (Fig. 2A and 2B), indicating that the various PAK3 shRNAs induced different pathways leading to cell death. The discrepancy between apoptosis induction and gene expression knockdown, as well as cell death stimulation, was also observed with SGK2 shRNA 2112 (Fig. 2C and 2D).

Autophagy is one of the main mechanisms for maintaining cellular homeostasis and is not directly associated with cell death, rather a self-cannibalisation pathway [30]; Patel et al., 2012). All shRNAs tested in our experiments, including four SGK2 shRNAs and the control shRNA (NT shRNA), induced autophagic marker LC3B conversion when compared with the no infection control (Fig. 2E). The three SGK2 shRNAs each displayed a stronger ability to induce autophagy in HeLa cells than NT shRNA while one (shRNA2112) behaved similarly to NT shRNA. SGK2 shRNA-induced autophagy marker (expression of LC3B) did not correlate with the decrease in cell number (compare Fig. 2E with 2D), also suggesting that the various SGK2 shRNAs mediated different pathways leading to cell death.

### PAK3 knockdown-deficient shRNA inhibition of HeLa cell growth

**Figure 3:**
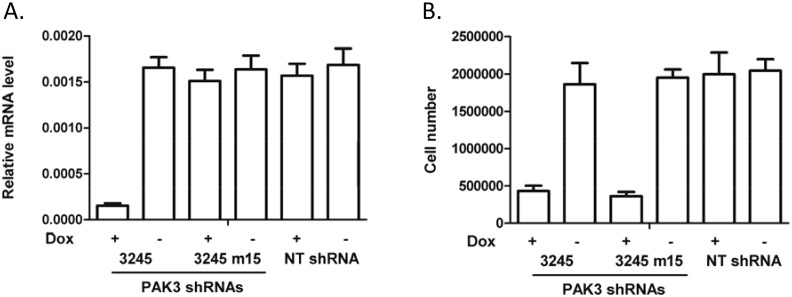
HeLa cell growth is not inhibited by a knockdown-deficient PAK3 shRNA. PAK3 mRNA levels were determined by quantitative reverse transcription PCR analysis at 72 hours after infection with the respective shRNA viruses. Live cell numbers were counted 5 days after the addition of 20 ng/mL doxycycline. The bar graph represents the average ± standard deviation of 3 replicates from one of two independent experiments with similar results. PAK3 mRNA levels were normalized with GAPDH expression; (A) Effect of wild-type (3245) and mutant PAK3 shRNA 3245 (3245 m15) on PAK3 mRNA levels in HeLa cells; (B) Effect of wild-type (3245) and mutant PAK3 shRNA 3245 (3245 m15) on HeLa cell growth.

To further clarify whether PAK3 shRNA-induced HPV+ cell death was associated with PAK3 knockdown, a PAK3 knockdown-deficient shRNA with a single mismatch to the PAK3 mRNA was tested for its ability to induce cell death in HeLa and CaSki cells. Addition of doxycycline to NT shRNA-infected HeLa cells did not decrease PAK3 mRNA expression nor affect cell growth (Fig. 3). A significant decrease in the PAK3 mRNA levels by doxycycline-induced expression of shRNA 3245 was observed, while PAK3 mRNA was not affected by doxycycline-induced expression of shRNA 3245 m15 (Fig. 3A). However, both wild-type and mutant PAK3 shRNAs dramatically inhibited HeLa cell proliferation/viability similarly (Fig. 3B), suggesting that HeLa cell death induced by PAK3 shRNA is not associated with PAK3 knockdown. A similar result was also observed with the CaSki cell line (data not shown).

### shRNAs that do not target human genes also inhibit proliferation/viability of HPV+ cervical cancer cells

**Figure 4:**
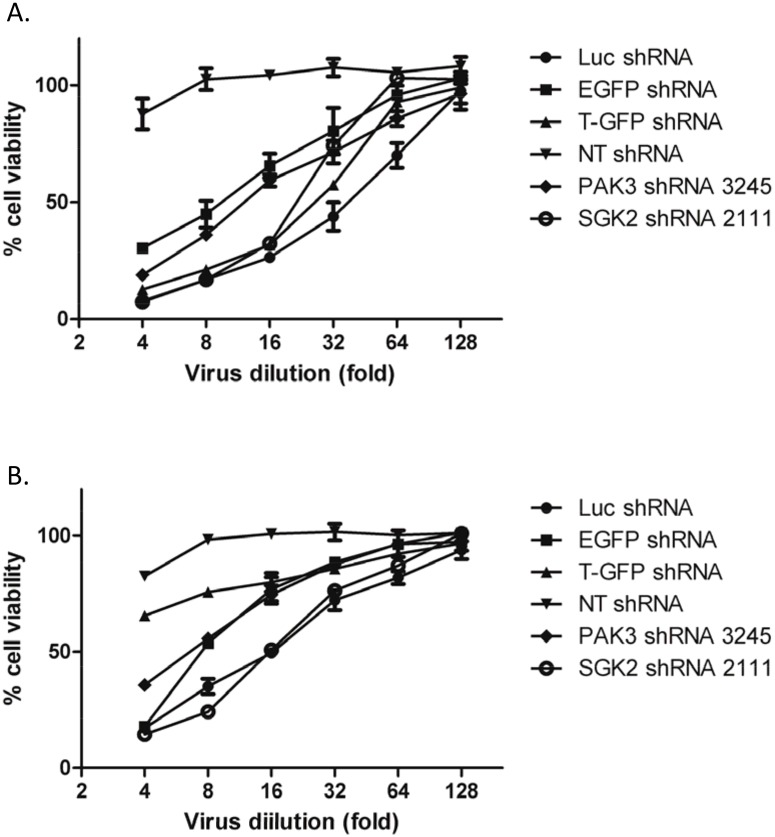
Inhibition of HPV+ cervical cancer cells by lentiviral shRNAs that do not target human genes. Cell viability was assessed using a CellTiter Blue assay 5 days after infection. Each of the viruses was titrated for infection to determine its potency of inducing HeLa cell proliferation/viability inhibition. Percent cell viability (fluorescence) in the presence of lentiviral shRNAs was calculated by comparing with a control lentivirus without shRNA expression. Data represent the average ± standard deviation of 4 replicates from one of three independent experiments with similar results; (A) Inhibition of HeLa cell proliferation/viability; (B) Inhibition of proliferation/viability of CaSki cells.

To determine whether HPV+ cervical cancer cells are specifically vulnerable to PAK3 and SGK2 shRNAs, we investigated four control lentivirus shRNA vectors that do not target human genes (Luc, EGFP, T-GFP and NT shRNAs, see Table 1) for their effect on the proliferation/ viability of HeLa and CaSki cells. No knockdown of PAK3 and SGK2 mRNA expression by the control shRNAs was observed (data not shown). Each virus was titrated to quantify its ability to induce inhibition of HeLa and CaSki cell proliferation/viability. PAK3 and SGK2 shRNAs were also included in the study for comparison. Three of the control shRNAs, except NT shRNA, suppressed HeLa cell growth with potencies comparable to PAK3 and SGK2 shRNAs (Fig. 4A). Two control shRNAs (Luc and EGFP shRNAs) induced growth inhibition of CaSki (Fig. 4B). Furthermore, HeLa and CaSki cell viability loss was observed using Luc and EGFP shRNAs in a dox-inducible shRNA expression vector (data not shown). Thus, HeLa and CaSki cells were shown to be susceptible to shRNAs that do not target human genes, suggesting that growth inhibition of these cells can be induced by shRNAs through non-specific gene pathways.

### Phenotypes induced by PAK3 and SGK2 shRNAs could not be rescued with the corresponding transgenic expressions

**Figure 5:**
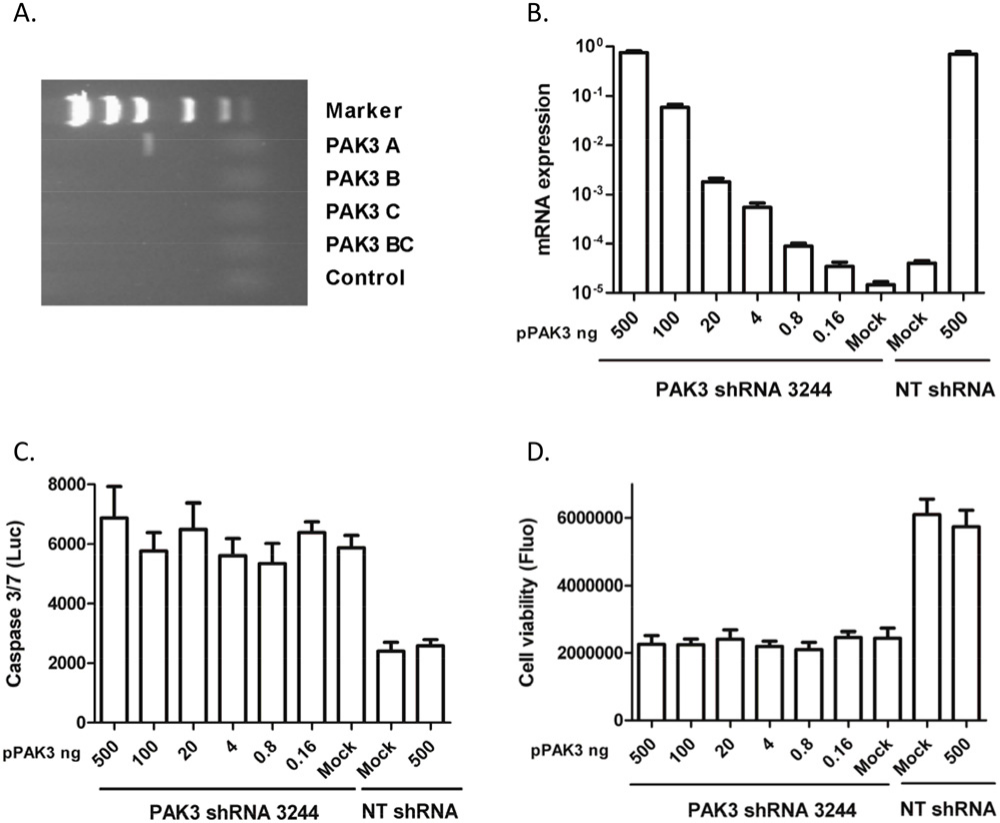
Rescue of PAK3 shRNA-induced phenotypes. (A) PAK3 variant A was identified in HeLa cells using a variant-specific RT-PCR method; (B) HeLa cells were transfected with serially diluted PAK3 expressing plasmids harboring silent mutations at the shRNA 3244 annealing site. Six hours after transfection, the cells were infected with PAK3 lentiviral shRNA 3244 that had been diluted 1:10. The lentiviral NT shRNA infection was used as a control. A quantitative PCR was performed to measure PAK3 mRNA 72 hours after infection. The bar graph presents quantities of PAK3 mRNA normalized using GAPDH mRNA. Data represent the average ± standard deviation of 4 replicates from one of three independent experiments with similar results; (C) HeLa cell apoptosis induced by PAK3 lentiviral shRNA 3244 was quantified with a caspase 3/7 glo luciferase (Luc) assay 72 hours after infection. Data represent the mean ± standard deviation of three independent experiments; (D) HeLa cell viability inhibition induced by PAK3 shRNA 3244 was determined using the CellTiter Blue assay 5 days after infection. Data represent the mean ± standard deviation of three independent experiments.

There are intrinsic limits on the specificity of RNAi, such as double-strand RNA-induced interferon response and off-target effects resulting from imperfect hybridization when a shRNA acts like a miRNA. An ideal experiment should demonstrate that expression of a mutated version of the targeted gene not recognized by the shRNA reverts or rescues the phenotype. As a first step, PAK3 expression was characterized in HeLa cells. Four PAK3 splice variants exist in mammals [31]. All of the variants are expressed in brain whereas in some tissues, such as kidney and liver, only PAK3 variant A is expressed [31]. By using the variant-specific RT-PCR method previously described [31], we confirmed that only variant A is expressed in HeLa cells (Fig. 5A). The cDNA coding region of PAK3 from HeLa cells is identical to GenBank sequence NM_002578. With respect to SGK2, its expression in HeLa cells was too low to be characterized; therefore, expression plasmids of the two reported SGK2 variants were used for the rescue study [19]. The PAK3 and SGK2 expression vectors harboring silent mutations at the corresponding shRNA hybridization sites were constructed (see Materials and Methods). These expressed mRNAs were resistant to PAK3 and SGK2 shRNA knockdown, respectively. HeLa cells were transfected with the knockdown-resistant PAK3 and SGK2 expressing vectors and then infected with lentivirus harboring the various shRNAs. The plasmid input was titrated for transfection to avoid artificial effects from protein over-expression.

After transfection of HeLa cells with serially diluted PAK3 plasmid harboring shRNA 3244-resistant mutations, PAK3 mRNA expression increased in a dose-dependent manner to the plasmid input, ranging from 1- to 18,000-fold over endogenously expressed PAK3 (Fig. 5B). Increase in PAK3 protein expression was observed in the transfected HeLa cells (S1 Fig.). No knockdown of the exogenous PAK3 mRNA by the shRNA was observed (Fig. 5B, compare 500 ng pPAK3 + PAK3 shRNA with 500 ng pPAK3 + NT shRNA). However, PAK3 shRNA 3244-induced HeLa cell apoptosis could not be rescued by the increasing transgenic expression of PAK3 (Fig. 5C). Transfection with the PAK3 expression plasmid did not prevent HeLa cell proliferation (Fig. 5D, compare pPAK3 transfected cells with mock) and did not rescue cell proliferation inhibition induced by the shRNA (Fig. 5D). Similar results were obtained from rescue experiments with two additional PAK3 shRNAs (shRNA 3245 and 5142) as well as for CaSki cells with the inducible PAK3 shRNA 3245 (data not shown).

**Figure 6:**
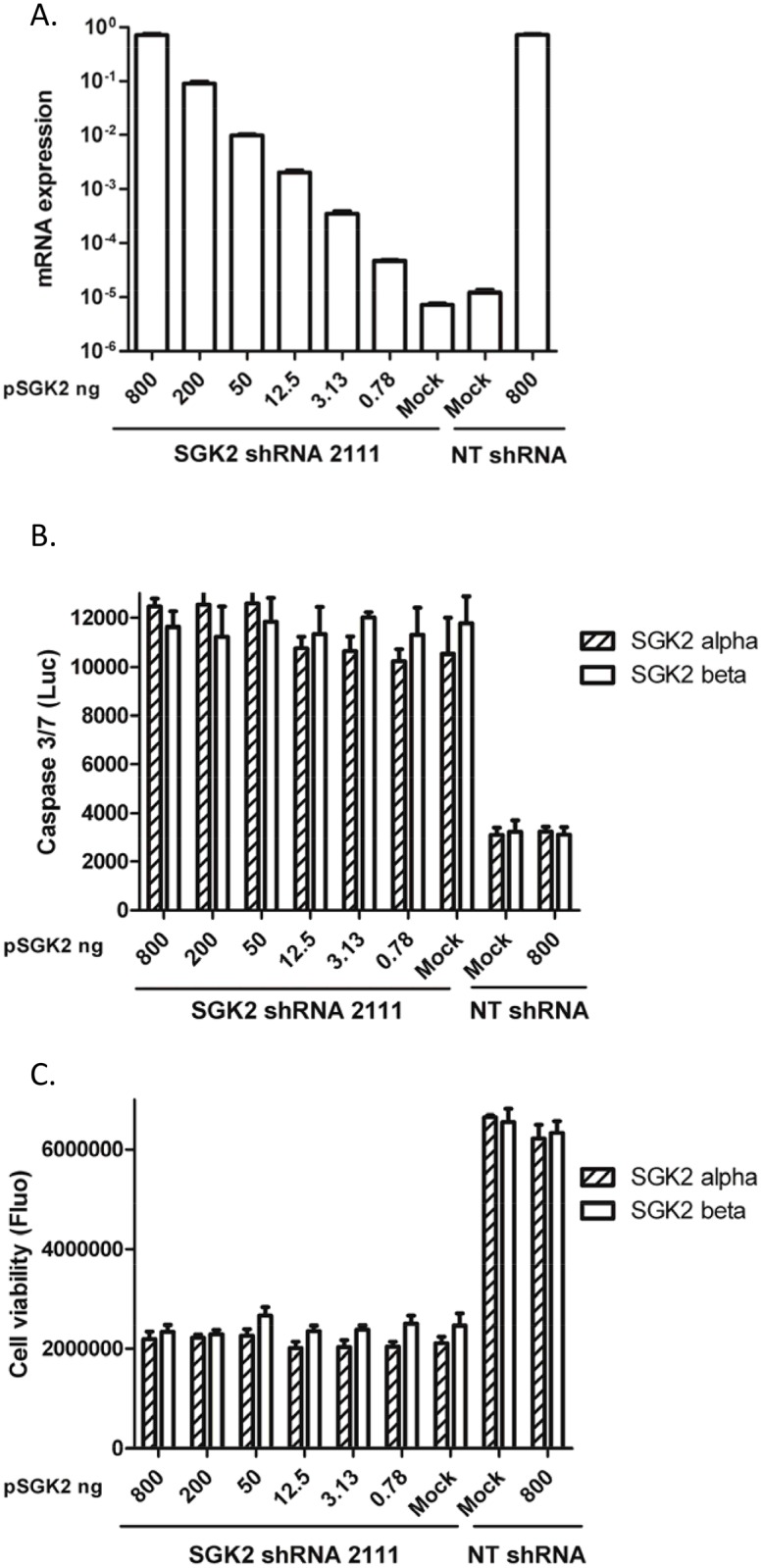
Rescue of SGK2 shRNA-induced phenotypes. (A) HeLa cells were transfected with serially diluted SGK2 expressing plasmids harboring silent mutations at the shRNA 2111 annealing site. Six hours after transfection, the cells were infected with SGK2 lentiviral shRNA 2111 (1:15 dilution). SGK2 mRNA expression levels were determined 72 hours after infection. The bar graph presents quantities of SGK2 mRNA normalized with GAPDH mRNA. Data represent the average ± standard deviation of 4 replicates from one of two experiments with similar results; (B) HeLa cell apoptosis induced by SGK2 shRNA 2111 was quantified with a caspase 3/7 glo assay 72 hours after infection. Two variants of SGK2 (alpha and beta) were used for the phenotype rescue analysis. Data represent the average ± standard deviation of two independent experiments; (C) HeLa cell proliferation/ viability inhibition induced by SGK2 shRNA 2111 was determined using the CellTiter Blue assay 5 days after infection. Data represents the average ± standard deviation of two independent experiments.

Two SGK2 variant plasmids were used for the rescue of SGK2 shRNA 2111-induced HeLa cell apoptosis and proliferation inhibition. SGK2 alpha mRNA expression increased 3- to 58,000-fold increase over endogenously expressed SGK2 72 hours after transfection (Fig. 6A). Increase in SGK2 protein expression in HeLa cells was also detected (data not shown). Transfection with pSGK2 did not significantly impact HeLa cell proliferation/viability but, as observed with the rescue experiments with PAK3, transgenic expression of either SGK2 alpha or beta did not rescue apoptosis and block growth inhibition of HeLa cells induced by SGK2 shRNA 2111 (Fig. 6B and C). Furthermore, transfection with the combination of SGK2 alpha and beta plasmids did not rescue the phenotypes (data not shown).

## Discussion

PAK3 and SGK2 were recently associated with the survival of HPV+ cervical cancer cells [10]. The authors found that the proliferation/viability of HPV+ cervical cancer cells, CaSki, HeLa and SiHa, was selectively inhibited by multiple PAK3 and SGK2 shRNAs. In a further study, the requirement of SGK2 and PAK3 for the survival of the HPV+ cervical cancer cells was attributed to the down-regulation of p53 in these cells. However, controls to rule out non-specific effects of these shRNAs and solid evidence to associate inhibition of cell proliferation with PAK3 or SGK2 expression knockdown were limited. Nevertheless, the suggested importance of PAK3 or SGK2 in the survival of HPV+ cervical cancer cells elevated our interest in these kinases as potential targets for HPV drug intervention. We have re-investigated the concept that PAK3 and SGK2 are essential to the survival of HPV+ cervical cancer cells by carefully examining the relationship between the shRNA-induced phenotypes and PAK3 or SGK2 knockdown, and found, in contradiction to the conclusions of Baldwin et al., that lethality of the PAK3 and SGK2 shRNAs to HeLa cells is not related to their knockdown of the corresponding gene expression but likely through off-target effects.

The PAK3 and SGK2 shRNAs used in our studies were validated for their capacity to induce a decrease in PAK3 or SGK2 expression in HeLa cells. Although PAK3 and SGK2 protein expression in HeLa cells was too low to be detected, the capacity of the various shRNAs to suppress PAK3 or SGK2 protein expression in cells highly expressing these proteins was confirmed. All tested PAK3 and SGK2 shRNAs inhibited the proliferation/viability of HeLa cells, consistent with the Baldwin’s study [10].

The Baldwin study reported that mechanisms of lethality in HeLa cells were different for the two kinases; SGK2 depletion caused autophagy, whereas PAK3 knockdown resulted in apoptosis, as shown by immunostaining of caspase 3 [10]. However, in our study, both PAK3 and SGK2 shRNAs induced apoptosis, as determined by a highly sensitive luciferase-based caspase 3/7 assay. The difference in these findings may be related to assay sensitivity. Autophagy or autophagocytosis, is a catabolic process involving the degradation of a cell′s own components through the lysosomal machinery. In the context of disease, autophagy has been seen as an adaptive response for cell survival, whereas in other cases, it appears to promote cell death and morbidity [32]. In our study, the autophagic LC3B marker formation was not associated with a decrease in HeLa cell number. Furthermore, the various shRNAs appeared to induce HeLa cell autophagy non-specifically since non-target shRNA produced similar quantities of LC3B as the SGK2 shRNAs.

There is a general consensus that different shRNAs targeting one gene should induce the same phenotypes through common pathways. In our study, the ability of PAK3 or SGK2 shRNAs to trigger HeLa cell apoptosis did not correlate with cell viability loss. This was clearly the case for PAK3 shRNA 3245 and SGK2 shRNA 2112, suggesting that these shRNAs eradicate HeLa cells through different pathways, which may not be target-specific. It should be noted that three of the non-human target control shRNAs (Luc, EGFP and T-GFP shRNAs) suppressed HeLa cell growth with potencies comparable to those of PAK3 and SGK2 shRNAs. The two control shRNAs (Luc and EGFP shRNAs) also induced growth inhibition of CaSki cells. To understand whether the p53 status was a determinant for the susceptibility of cancer cells to PAK3 or SGK2 knockdown, we tested multiple p53 wild type and mutant cancer cell lines, including the HPV-negative cervical cancer cell line, C33A^p53mut^, melanoma A375^p53wt^, lung cancer A549^p53wt^, colon cancer HCT 116^p53wt^, liver cancer HepG2^p53wt^, and osteosarcoma U2OS^p53wt^. In general, the viability of these cell lines was not affected by the presence of PAK3 or SGK2 shRNAs although A375^p53wt^ was sensitive to SGK2 shRNAs and A549^p53wt^ was sensitive to PAK3 shRNAs (data not shown). Thus, the susceptibility of cancer cells to knockdown by PAK3 or SGK2 shRNAs was not p53 function-dependent but rather cell line-dependent.

When performing RNAi experiments, it is important to use several shRNAs targeting different regions of the same mRNA that produce the same phenotype to exclude non-specific effects. [33, 34], However, for relatively common phenotypes (e.g., slower growth, apoptosis and developmental arrest), the use of two or more different RNAi inducers is not sufficient to decipher whether the phenotype is specific to the down-regulated gene [34]. In such a case, a rescue experiment is highly desirable [32]. The ultimate control for any RNAi experiment is rescue by expression of the target gene in a form that is refractory to the shRNA of interest. Therefore, we carefully designed a phenotype rescue study with the appropriate positive and negative controls to verify the roles of PAK3 and SGK2 in shRNA-induced apoptosis and viability loss of HeLa cells. No phenotype reversion by the supplemental expression of PAK3 or SGK2 was observed in the rescue studies with three PAK3 and three SGK2 shRNAs. Similar results were observed in a rescue study using the CaSki cell line. Thus, lethality of the PAK3 and SGK2 shRNAs to HeLa or CaSki cells was not a consequence of inhibition of PAK3 and SGK2 expression. This conclusion was supported by results from an additional study employing a PAK3 shRNA 3245 mutant whose mRNA knockdown capacity was completely abrogated by introducing a single mutation to its 3’ seed region. However, the “dud” shRNA still inhibited HeLa and CaSki cell growth to a similar extent as the wild type PAK3 shRNA 3245.

In summary, we have validated the function of PAK3 and SGK2 lentiviral shRNAs in the knockdown of PAK3 and SGK2 gene expression, and also observed viability loss of HPV+ cervical cancer cells (HeLa and CaSki) mediated by the lentiviral shRNAs. However, we were unable to demonstrate a correlation between the death of HPV-transformed cells with PAK3 or SGK2 knockdown in rescue studies, the gold standard for RNAi phenotype validation. We also observed that HPV+ cervical cancer cells were sensitive to shRNAs that do not target human genes. Therefore, the lethality of PAK3 or SGK2 shRNAs to HPV+ cervical cancer cells did not appear related to the inhibition of PAK3 or SGK2 expression but rather through a non-specific RNAi effect.

## Supporting Information

S1 Fig PAK3 protein expression. HeLa was transfected with serially diluted PAK3 mutant or wild type-expressing plasmids and then infected with shRNA-expressing lentivirus. PAK3 protein expression was measured by Western immunoblot with a PAK3 monoclonal antibody (N-19) 72 hours after infection. Abundantly expressed PAK2 protein migrated with PAK3 protein in HeLa cells (evidenced by PAK2 knockdown study; data not shown), and the PAK3 antibody N19 had cross-reactivity with PAK2.
(TIF)

S2 Fig SGK2 protein expression. HeLa cell was transfected with serially diluted SGK2 mutant or wild type-expressing plasmids and then infected with SGK2 shRNA 1648. SGK2 protein expression was measured by Western immunoblot with a SGK2 monoclonal antibody (3Q-2) 72 hours after infection.
(TIF)
