# Supplementary figures and images for: Lethality of PAK3 and SGK2 shRNAs to Human Papillomavirus Positive Cervical Cancer Cells Is Independent of PAK3 and SGK2 Knockdown
Source: PLoS One. 2015 Jan 23;10(1):e0117357. doi: 10.1371/journal.pone.0117357 (PMC4304782; doi:10.1371/journal.pone.0117357)

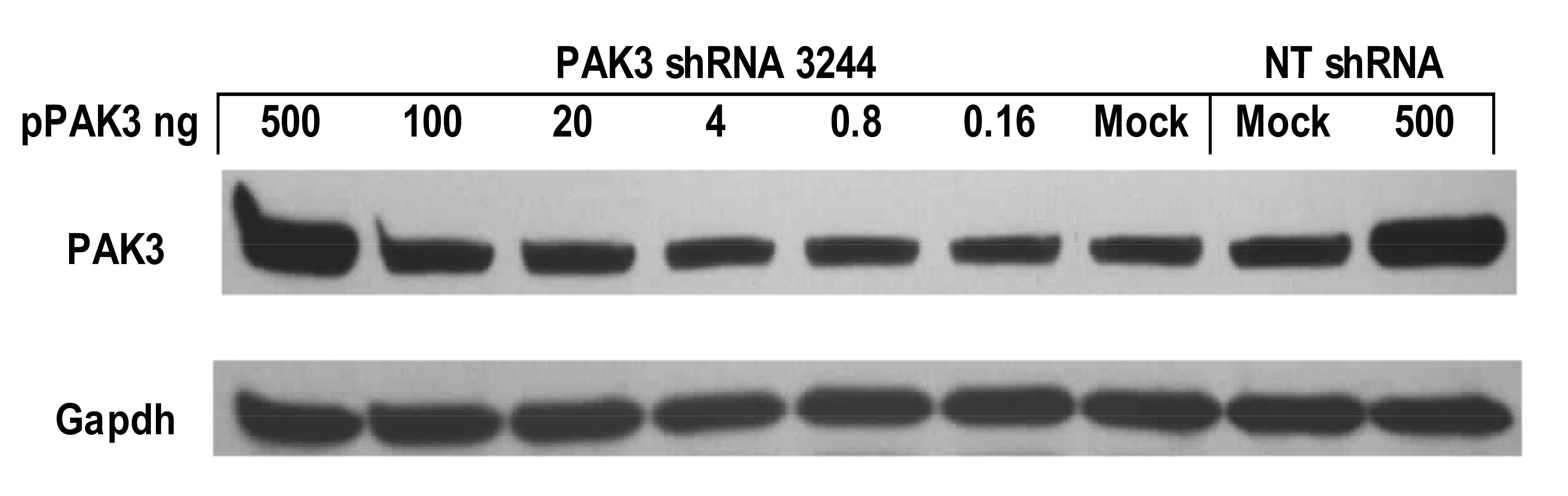

Supplement: S1 Fig — HeLa was transfected with serially diluted PAK3 mutant or wild type-expressing plasmids and then infected with shRNA-expressing lentivirus. PAK3 protein expression was measured by Western immunoblot with a PAK3 monoclonal antibody (N-19) 72 hours after infection. Abundantly expressed PAK2 protein migrated with PAK3 protein in HeLa cells (evidenced by PAK2 knockdown study; data not shown), and the PAK3 antibody N19 had cross-reactivity with PAK2. (TIF) [file pone.0117357.s001.tif]

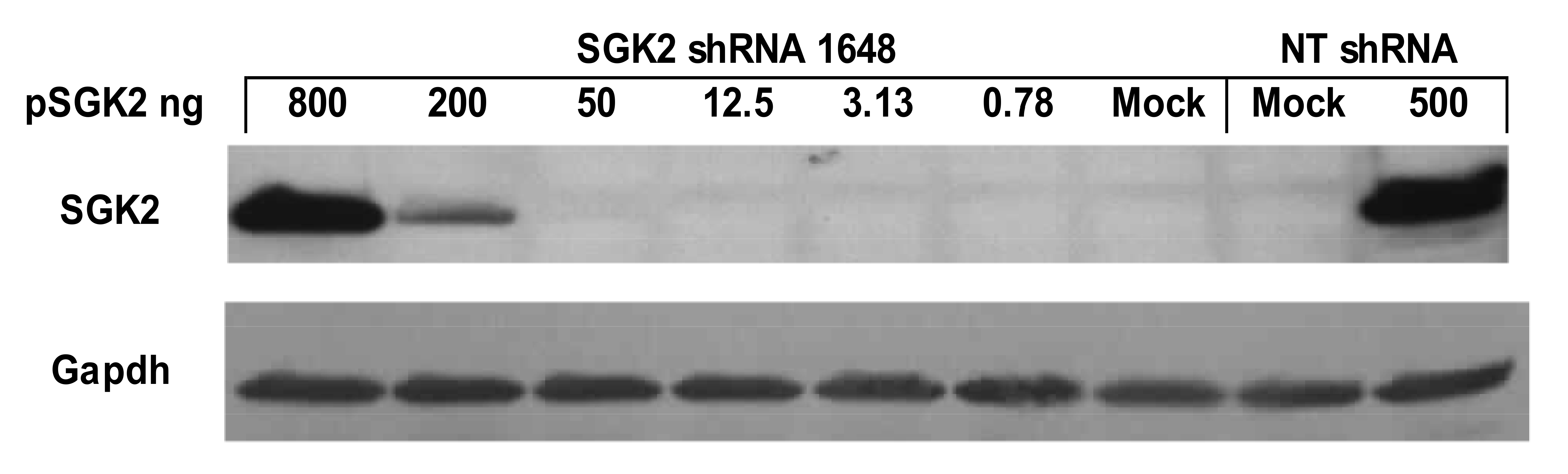

Supplement: S2 Fig — HeLa cell was transfected with serially diluted SGK2 mutant or wild type-expressing plasmids and then infected with SGK2 shRNA 1648. SGK2 protein expression was measured by Western immunoblot with a SGK2 monoclonal antibody (3Q-2) 72 hours after infection. (TIF) [file pone.0117357.s002.tif]
